# Does the primate face cue personality?

**DOI:** 10.1017/pen.2023.5

**Published:** 2023-08-09

**Authors:** Vanessa A D Wilson, Michaela Masilkova

**Affiliations:** 1 Department of Comparative Cognition, Institute of Biology, University of Neuchâtel, Neuchâtel, Switzerland; 2 Department of Comparative Language Science, University of Zurich, Zurich, Switzerland; 3 Center for the Interdisciplinary Study of Language Evolution (ISLE), University of Zurich, Zurich, Switzerland; 4 Department of Game Management and Wildlife Biology, Faculty of Forestry and Wood Sciences, Czech University of Life Sciences, Prague, Czech Republic

**Keywords:** fWHR, dominance, rank, evolution, social signal, sexual selection, perception

## Abstract

When looking at others, primates primarily focus on the face – detecting the face first and looking at it longer than other parts of the body. This is because primate faces, even without expression, convey trait information crucial for navigating social relationships. Recent studies on primates, including humans, have linked facial features, specifically facial width-to-height ratio (fWHR), to rank and Dominance-related personality traits, suggesting these links’ potential role in social decisions. However, studies on the association between dominance and fWHR report contradictory results in humans and variable patterns in nonhuman primates. It is also not clear whether and how nonhuman primates perceive different facial cues to personality traits and whether these may have evolved as social signals. This review summarises the variable facial-personality links, their underlying proximate and evolutionary mechanisms and their perception across primates. We emphasise the importance of employing comparative research, including various primate species and human populations, to disentangle phylogeny from socio-ecological drivers and to understand the selection pressures driving the facial-personality links in humans. Finally, we encourage researchers to move away from single facial measures and towards holistic measures and to complement perception studies using neuroscientific methods.

Top-down approaches to primate personality offer insights into the evolution and diversity of personality traits within the primate order (Weiss, 2017). Comparative approaches also allow us to address interspecific differences in endocrinological (Wilson, Guenther, Øverli, Seltmann, & Altschul, 2019), genetic (von Borell, Weiss, & Penke, 2019) and physical phenotypic correlates of behaviour (Kern, Robinson, Gass, Godwin, & Langerhans, 2016). Such comparison is key to understanding variance in fitness outcomes such as stress responses, reproductivity and survival (Blaszczyk, 2020). Within this framework, signal^1^ strength is an important variable, since social signals are determinants of partner preference and combative encounters, which can affect fitness.

Amongst primates, faces are an important consideration for signalling, as they provide a wealth of information that can help inform social decisions. Primate faces are complex in terms of shape, colouration and the presence of facial hair, and all these components might be relevant for signalling (Waller, Kavanagh, Micheletta, Clark, & Whitehouse, 2022). Dynamically, faces can express emotions and provide other information (Waller, Whitehouse, & Micheletta, 2017), which in primates can be used to mediate exchanges across the dominance hierarchy, such as using fear grins to show submissive (Maestripieri & Wallen, 1997) and affiliative (Waller & Dunbar, 2005) behaviour. Static faces can communicate information about fertility and fitness, such as reproductive-state-related changes in skin redness of rhesus macaques (*Macaca mulatta*), with male seasonal colouration changes linked to female mate preference (Dubuc, Allen, Maestripieri, & Higham, 2014). They have also been found to advertise dominance, via facial colouration in mandrills (*Mandrillus sphinx*) (Setchell, Smith, Wickings, & Knapp, 2008) and canine size in baboons (*Papio* sp.) (Galbany, Tung, Altmann, & Alberts, 2015). Thus, primate faces appear to contain multi-component information that is not only used in direct communication but also can indicate fitness to potential mates and competitors via sexually selected traits.

One topic that has been much debated in the human literature is the link between dominance and facial morphology. Starting with the work of Weston, Friday, Johnstone, and Schrenk (2004), who found an inverse relationship between canine height sexual dimorphism and bizygomatic width dimorphism amongst primates, the hypothesis emerged that in some primate species, dimorphism of facial width evolved as a result of intra-sexual selection by male–male competition (Weston et al., 2004, Weston, Friday, & Liò, 2007), whereby a wider zygomatic arch provides a combat advantage via skull strength (Lefevre et al., 2014; Stirrat, Stulp, & Pollet, 2012). This was supported by multiple human studies linking male facial width, typically measured as facial width-to-height ratio (fWHR, Fig. 1a) (Weston et al., 2007), to dominance (mostly self-reported or inferred, but also estimated from military rank and economic game outcomes) (Geniole, Denson, Dixson, Carré, & McCormick, 2015; Polo et al., 2022) and aggression (Goetz et al., 2013; Haselhuhn, Ormiston, & Wong, 2015; Stirrat et al., 2012; Třebický, Havlíček, Roberts, Little, & Kleisner, 2013; Wen & Zheng, 2020; Zilioli et al., 2015). Wider faces are also perceived as more dominant (Alrajih & Ward, 2014; Lefevre & Lewis, 2014; Mileva, Cowan, Cobey, Knowles, & Little, 2014), acting as cues or even signals of individual dominant/aggressive personality traits.

**Figure 1.**
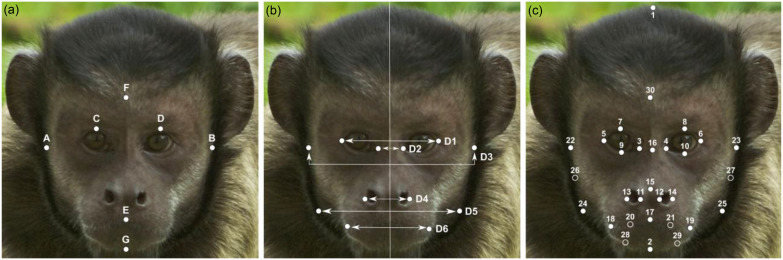
Positions of landmarks for morphometric measurements: a. fWHR (A–B)/[midpoint(C,D)–E], fLHFH [midpoint (C,D)–G]/(F–G), fWLFH (A–B)/[midpoint(C,D)–G] according to Wilson et al. (2014); b. asymmetry calculated as the absolute difference from the midpoint of lines D1-D6 according to Little et al. (2012); c. set of 30 landmarks (full circles) and semi-landmarks (open circles) (for a description, see Table S1 in Supplementary materials) delineating facial features (adapted for nonhuman primates from the set of landmarks designed for humans; e.g., Kleisner et al., 2019). *Abbreviations:* fWHR *=* facial width-to-height ratio, fLHFH *=* facial lower-height/full-height, fWLFH *=* face width/lower face height.

However, the results of studies of facial-dominance links in humans are often inconclusive and impeded by methodological issues. Firstly, several null findings are inconsistent with the proposed link between bizygomatic width and dominance (Özener, 2012; Wang, Nair, Kouchaki, Zajac, & Zhao, 2019), including mixed findings for facial perceptions (Durkee & Ayers, 2021) and sexual dimorphism of facial morphology (Kramer, 2017; Lefevre et al., 2012; Penton-Voak et al., 2001; Summersby, Harris, Denson, & White, 2022; Wen & Zheng, 2020), as well as limited evidence linking facial width to combat success (Carré & McCormick, 2008; Stirrat et al., 2012; Třebický et al., 2013; Zilioli et al., 2015). Secondly, the underlying mechanisms that could drive this relationship are poorly understood. There is little evidence for testosterone providing concurrent effects on craniofacial growth and behavioural traits (Bird et al., 2016; Eisenbruch, Lukaszewski, Simmons, Arai, & Roney, 2017; Hodges-Simeon, Sobraske, Samore, Gurven, & Gaulin, 2016; Kordsmeyer, Freund, Pita, Jünger, & Penke, 2019). Thirdly, the use of ratio measures can be problematic, leading to spurious correlations (Kronmal, 1993). Fourthly, most research on facial dimorphism and links to behaviour or personality remains in the human domain. Expanding this to comparative work could help us understand which selection pressures might drive facial cues of dominance or other traits (Wilson, Weiss et al., 2020).

In the remainder of this paper, we discuss studies conducted in nonhuman primates on this topic: focusing on static faces; discussing the potential selection pressures and underlying mechanisms; and making proposals for future research that could benefit a broader understanding of morphological cues to personality.

## 1. Facial morphology and behavioural correlates in nonhuman primates

Early studies on apes, focusing predominantly on cranial measurements, have revealed varying levels of sexual dimorphism. Gorillas (*Gorilla gorilla*, subspecies not specified) exhibit the strongest cranial sexual dimorphism amongst hominids (excluding bonobos, which were not tested) with low dimorphism in chimpanzee skull morphology (O’Higgins & Dryden, 1993). Orangutans also display strong sexual dimorphism in cranial morphology, which begins in infancy, with males exhibiting a continuous growth of the zygomatic bone from early adolescence into adulthood (Hens, 2005). Male orangutans also develop cheek flanges, secondary sexual characteristics that are linked to androgen levels (Marty et al., 2015), and silverback gorillas develop sagittal crests of fatty and fibrous tissue (Breuer, Robbins, & Boesch, 2007). These sexually dimorphic features are correlated with their reproductive success (Banes, Galdikas, & Vigilant, 2015; Breuer, Robbins, Boesch, & Robbins, 2012).

More recent studies have focused on facial morphological links to behaviour, specifically to personality traits (individual behavioural characteristics consistent across time; capitalised further in the text, e.g., Dominance) and to characteristics of dominance hierarchies (relative ranking of individuals dependent on the outcomes of their present agonistic interactions; labelled further in the text with lowercase, e.g., dominance). Personality structures of nonhuman primates assessed by trait rating (specifically using the Hominoid Personality Questionnaire; Weiss, 2017), unlike the human five-factor model (McCrae & Costa, 1987), include a separate Dominance-related personality dimension (labelled as Assertiveness or Dominance depending on the species, see Table 1). This separate Dominance-related personality dimension, indicating dominant and often aggressive social tendencies (except for Assertiveness in bonobos reflecting affiliative dominant tendencies; Martin, Staes, Weiss, Stevens, & Jaeggi, 2019), evolved as a result of the central role of dominance interactions in nonhuman primate social interactions (Weiss, 2022). Personality structures of some species, however, include more than one Dominance-related dimension. Similar to chimpanzees (Weiss et al., 2023), rhesus macaque Assertiveness is related to dominance in social interactions (Kohn et al., 2016), whilst Confidence refers to confidence in the presence of environmental or social stressors (Adams et al., 2015). The characteristics of dominance hierarchies, assessed by observing outcomes of agonistic interactions, are usually expressed as rank (called “dominance status” in Altschul, Robinson, Coleman, Capitanio, & Wilson, 2019; “alpha status” in Lefevre et al., 2014; “agonistic dominance” in Martin et al., 2019).

**Table 1.**
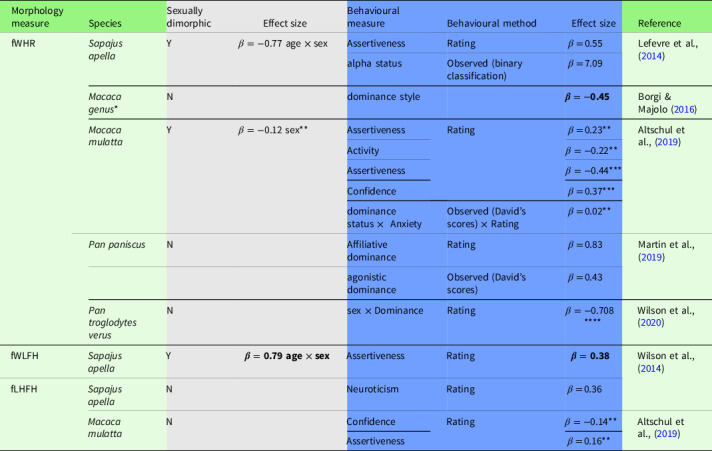
Morphological facial features in nonhuman primates and correlates with behaviour

*Note*. Facial morphology measures (see Fig. 1a for definition) were taken from facial photographs. Grey cells indicate the effect size for sexual dimorphism. Blue cells indicate effect size for behavioural measures. Values in bold indicate where coefficients were calculated from raw data. Rating of personality traits was conducted using the Hominoid Personality Questionnaire (Weiss, 2017). Results were only significant in individuals:

*Data represent 9 species of the *Macaca* genus.

**<8 years,

***>8 years,

****in females.

In brown capuchins (*Sapajus apella*), fWHR is sexually dimorphic, with higher ratios for males and alpha individuals, and correlates with the personality component Assertiveness (Lefevre et al., 2014). Alpha males in particular are observed to have bulkier facial features, an effect that appears to be driven by direct access to females (Paukner et al., 2021). Higher fWHR was associated with despotic social style in the *Macaca* genus (Borgi & Majolo, 2016) and with Assertiveness in immature rhesus macaques (Altschul et al., 2019). fWHR has also been associated with Assertiveness (reflecting affiliative dominance) and rank (reflecting agonistic dominance) in bonobos (*Pan paniscus*) (Martin et al., 2019) and with Dominance in female chimpanzees (*Pan troglodytes verus*) (Wilson, Weiss et al., 2020), yet for all these species (macaques, bonobos and chimpanzees) fWHR is not sexually dimorphic once accounting for sexual dimorphism in body size.

Several other measures, which have been found to be sexually dimorphic in humans (Penton-Voak et al., 2001), have also been assessed in nonhuman primates: face width/lower face height (Fig. 1a) has been linked to Assertiveness in brown capuchins and facial lower-height/full-height (Fig. 1a) to Neuroticism in brown capuchins (Wilson et al., 2014) and to lower Confidence in immature rhesus macaques (Altschul et al., 2019).

To summarise, personality, in particular Dominance-like traits, seems to have links to facial metrics in all species studied, but these relationships depend on age, sex and even subspecies (see Table 1), suggesting divergent selection pressures driving facial cues to Dominance. Together, these findings paint an interesting but varied picture of the role of facial morphology in primate behaviour, suggesting that human studies have only just scratched the surface of a potentially much broader field. The available studies on facial-personality links in primates, however, are not without limitations:

1. So far, the studied species represent a limited pool of the behavioural diversity found amongst primates, so potential socio-ecological drivers of interspecific differences cannot be untangled from phylogenetics.
2. Metric-based results may vary depending on whether measures are based on skull measurements or taken from photographs of living subjects (Kramer, 2017). Measures of skulls can be informative of sex-dependent growth trajectories independent of adiposity, but they do not account for facial characteristics that emerge in none-bone tissue, such as the secondary sexual characteristics found in gorillas and orangutans.
3. To date, most studies have focused on behavioural links to single, ratio-based measures (Fig. 1a).

## 2. The role of sexual selection in evolution of facial-personality links

Research linking the fWHR and facial lower-height/full-height to socially relevant Dominance-related personality traits (e.g., Dominance, Assertiveness, Neuroticism) suggests the potential role of static facial features as a cue in intraspecific communication (Table 1), which is long-lasting and available to many bystanders (compared to signalling through short-term facial expressions aimed at one or a few bystanders; Petersen & Higham, 2020). Sexual selection, including inter- and intra-sexual selection, has been hypothesised to generate and maintain facial-personality links (Lefevre et al., 2014; Petersen & Higham, 2020; Wilson, Weiss et al., 2020).

Intra-sexual selection acts on morphological or behavioural traits that increase an individual´s competitive ability. In primates (Table 1) including humans (e.g., Geniole et al., 2015; Haselhuhn et al., 2015), fWHR and potentially other facial features might act as badges of status (Bergman & Sheehan, 2013; Petersen & Higham, 2020), reflecting status-seeking motivations, Dominance-related personality traits and fighting ability with the potential role of modulating social interactions. Indeed, evidence that faces of men are wider during early sexual maturity, compared with faces of older men, suggests that fWHR plays a role in intra-sexual competition (Summersby et al., 2022).

Inter-sexual selection acts on traits that improve individual reproductive success. For example, western lowland gorilla males with larger crests have a higher number of females (Caillaud, Levréro, Gatti, Ménard, & Raymond, 2008) and better reproductive success (Breuer et al., 2012). This suggests that certain sexually dimorphic cranial features are primarily under inter-sexual selection. In general, morphological traits reflecting individual quality either as a prospective partner or parent (direct benefits) or in terms of good genes (indirect benefits) are used as signals in mate choice. Experimental studies on birds and fish demonstrated that females consider their partner’s personality (e.g., Boldness or Exploration) as a signal of their quality in mate choice and that personality is often linked with variation in reproductive success (reviewed in Schuett, Tregenza, & Dall, 2010). Hence, any facial feature reflecting personality traits relevant to resource provision and paternal care (potentially Extraversion or Agreeableness) or protection from infanticide (potentially Assertiveness or Dominance) might be used as a cue by potential mates (Martin et al., 2019; Weston et al., 2004). In primates, for example, facial symmetry (Fig. 1b) is perceived by partners as attractive and might be used as a cue to indirect benefits, such as good genes, better health and condition (Little, Paukner, Woodward, & Suomi, 2012; Sefcek & King, 2007; Waitt & Little, 2006). Recent studies, however, found no significant association between attractiveness and facial symmetry in any of the tested human samples (Kleisner et al., 2017; Kočnar, Saribay, & Kleisner, 2019; Van Dongen & Gangestad, 2011). Hence, our understanding of face as a cue to personality and its role in mate choice in primates is limited.

## 3. Selection pressures driving facial-personality links

Unlike facial lower-height/full-height, which is associated with diverse personality traits across primates (Table 1) and thus might have diverged since the split of Catarrrhines and Platyrrhines, the link between fWHR and Dominance-related personality traits might predate this divergence. Yet, the pattern of associations between fWHR and Dominance-related personality traits in primates is not universal. Various species- or sex-specific selection pressures might explain these diverse patterns. First, provided that the face is a badge of status signalling personality characteristics relevant to choosing a partner or avoiding conflict escalation, it is expected to be found in primates living in large or dynamic groups, or where interactions with out-group members are frequent; in such scenarios, individual recognition is either limited or absent, and judgement based on first impression is crucial to fitness (Bergman & Sheehan, 2013; Grueter, Isler, & Dixson, 2015). This might be further affected by sex-specific natal dispersal patterns – for example, facial-personality links might be more critical in the dispersing sex.

Second, the species-specific expression of dominance and a level of competitiveness might have affected the nature of personality traits linked to facial features and the strength of the correlation. In bonobos, in which social status is achieved by affiliative behaviour and coalitionary support, fWHR was more strongly related to Assertiveness (reflecting affiliative dominant tendencies) rather than rank (based on agonistic interactions) (Table 1). Contrastingly, macaque species with a despotic dominance style, for example, rhesus macaques, have larger fWHR than more tolerant species, for example, crested macaques (*Macaca nigra*) (Borgi & Majolo, 2016). Humans are considered to have evolved from egalitarian societies (Boehm, 1999; Kaplan, Hooper, & Gurven, 2009), as evidenced by their high social tolerance for out-group members, and reflected in their personality structure: compared to other primates, humans do not have a separate Dominance personality dimension (Weiss, 2022). These hierarchical differences could explain why links between fWHR and Dominance-related personality traits are relatively weaker than those in chimpanzees or capuchin monkeys (Table 1) (Wilson, Weiss et al., 2020).

Third, the sex-specific patterns of facial-personality links might be attributed to the sex-specific dominance strategies and rank stability. Whilst in humans the fWHR-dominance link is found only in males, because human males exhibit dominant behaviour and aggression more than females (Archer, 2004), in capuchins and bonobos, this link was found in both sexes (Table 1), as both sexes express similar levels of dominance (Gazes, Schrock, Leard, & Lutz, 2022; Stevens, Vervaecke, de Vries, & van Elsacker, 2007). Female chimpanzees exhibiting relatively stable rank across the lifespan may rely on signalling by facial morphological features compared to males, who experience dynamic changes in rank across the lifespan and depend on signalling by facial expressions and aggressive behaviour (Wilson, Weiss et al., 2020).

Finally, the mating system might act as another selection pressure. Faces of polygynous primates were rated by human raters as more masculine on a 6-point rating scale (based on presence of sexually dimorphic features, such as differences in pelage) than monogamous and promiscuous primates (A. Dixson, Dixson, & Anderson, 2005). A similar pattern is also expected in terms of facial-personality links. These explanations are of course not mutually exclusive, and several selection pressures might have acted simultaneously.

## 4. Proximate mechanisms underlying facial-personality link

Because the research on facial morphological cues to personality in nonhuman primates is relatively new, the direct causal links have not yet been studied. Hence, we must mainly rely on studies of the proximate mechanisms of human facial morphology. In this section, we will discuss two possible candidate mechanisms: neuroendocrine mechanisms and fluctuations during development.

Across vertebrate species, gonadal steroid hormones play a crucial role in bone growth (Gandelman, Simon, & McDermott, 1979; Juul, 2001; Whitehouse et al., 2015) and sex differentiation of behaviour (Berenbaum & Beltz, 2011; Sisk & Zehr, 2005; Thornton, Zehr, & Loose, 2009). Since fWHR is linked to aggressive and dominant tendencies in primates including humans, testosterone exposure during pregnancy and puberty has been hypothesised to have critical organisational effects on morphology and neural structure, the latter causing behaviour differentiation. This was confirmed by a recent study on humans, documenting that newborns with higher umbilical cord testosterone levels had more masculine faces (measured as gender score) as adults (Whitehouse et al., 2015). Surprisingly, this study did not find an association between prenatal testosterone levels and adult fWHR. In rhesus macaques, the experimental prenatal administration of testosterone masculinised juvenile and adult sexual behaviour (reviewed in Thornton et al., 2009). The mediating effect of testosterone, however, may not be universal in primates. Instead, inter-species variability is expected due to the species- and sex-specific selection pressures. For example, the second-to-fourth digit ratio, a proxy of prenatal testosterone levels, was lower (higher prenatal testosterone levels) in polygynous primate species and species with high levels of intra-sexual competition compared to monogamous and polyandrous species and those with low levels of intra-sexual competition (Nelson & Shultz, 2009).

The sex differentiation of morphology and behaviour established prenatally is further strengthened during puberty (Marečková et al., 2011). Adult circulating baseline or reactive testosterone levels have no (Bird et al., 2016) or minimal effect (Lefevre, Lewis, Perrett, & Penke, 2013) on fWHR. Regardless, the organisational effects of prenatal and pubertal testosterone exposure on behaviour and face morphology proved to be permanent and hence might also underlie the facial-personality links. Testosterone, however, might not be the only neuroendocrine mechanism underlying the facial-personality links. A study on lemurs, which are characterised by masculinisation of female genitalia and female dominance over males, concluded that the testosterone:oestrogen ratio rather than absolute prenatal testosterone levels promoted female masculinisation (Ostner, Heistermann, & Kappeler, 2003). The prenatal testosterone:oestrogen ratio has been documented to contribute to the behavioural sex differentiation also in humans (Mitsui et al., 2019). Finally, the effects of steroid hormones are often moderated by interaction with other mechanisms, such as cortisol (Carré & Archer, 2018), growth hormone (Marečková et al., 2011), and androgen sensitivity caused by variation in a number of tri-nucleotide (CAG) repeats in the androgen receptor gene (Simmons & Roney, 2011).

Instability during critical phases of foetal and postnatal development caused by environmental (e.g., parasite load, resources quality and quantity, diseases) or genetic (e.g., homozygosity, inbreeding, mutation) stressors may result in fluctuating asymmetry (Caccavo, Lemos, Maroja, & Gonçalves, 2021; Parsons, 1992, but see also Lens, Dongen, Kark, & Matthysen, 2002). Fluctuating asymmetry refers to minor random deviations from perfect symmetry (Valen, 1962) and can be expressed as individual variability in the asymmetry of craniofacial features (Caccavo et al., 2021; Fig. 1b) and other bilaterally symmetrical structures (Kucheravy, Waterman, & Roth, 2022). Facial asymmetry in humans has been found to be associated with poor health, specifically number of respiratory diseases and their duration (Thornhill & Gangestad, 2006, but see Rhodes et al., 2001), and personality traits, such as Extraversion, Agreeableness, Neuroticism or Assertiveness (Borráz-León & Cerda-Molina, 2015; Fink, Neave, Manning, & Grammer 2005; Holtzman, Augustine, & Senne, 2011; Pound, Penton-Voak, & Brown, 2007), although the reported associations are usually weak or inconsistent (Hope et al., 2011; Van Dongen & Gangestad, 2011) or show null associations with attractiveness (Kleisner et al., 2017; Kočnar et al., 2019). Moreover, stress might be theoretically dependent on the rank and personality of the mother as, for example, subordinate, less confident or more sociable females face a higher risk of injury or pathogen transmission (Robinson et al., 2018). Investigating the potential links between facial symmetry and personality might be a promising avenue.

## 5. Does the primate face provide cues to personality?

Numerous studies reveal that humans perceive personality differences in conspecific faces (Kramer & Ward, 2010; Little & Perrett, 2007), including dynamic features (Kavanagh, Whitehouse, & Waller, 2022), and even in other species (Clark, Butler, Ritchie, & Maréchal, 2020; Kramer & Ward, 2012). In parallel, there is a large body of comparative research on how people and nonhuman primates look at faces, from what features they attend to (Dahl, Wallraven, Bülthoff, & Logothetis, 2009; Gothard, Erickson, & Amaral, 2004; Kano, Call, & Tomonaga, 2012), to holistic processing (Carp et al., 2022; Parr, 2011; Wilson, Kade et al., 2020). Yet, little is known about whether and how nonhuman primates perceive different facial cues to personality traits.

Studying how individuals perceive information encoded in facial features of conspecifics is key to understanding whether personality traits can be read from faces. The simplest way to test this is using the looking time paradigm, which assesses gaze responses to two different stimuli (Wilson, Bethell, & Nawroth, 2023). Numerous studies have used this approach to examine whether primates differentiate between images of faces that depict varying social information (Wilson et al., 2023; Winters, Dubuc, & Higham, 2015), such as differences in fitness (indicated by skin redness) (Waitt et al., 2003), age (Almeling, Hammerschmidt, Sennhenn-Reulen, Freund, & Fischer, 2016) and emotion expression (Pritsch, Telkemeyer, Mühlenbeck, & Liebal, 2017). Such studies suggest that being able to attend to socially relevant information is a potentially universal feature amongst primates.

Yet, to what extent different species advertise socially relevant traits in facial features, or use this information to inform social interactions, is largely unknown. One study found that long-tailed (*Macaca fascicularis*) and rhesus macaques can distinguish between human faces based on features depicting Trustworthiness (Costa et al., 2018). Moreover, both monkey and human participants exhibited reduced-looking durations to faces with higher fWHRs. Male rhesus macaques also demonstrate rank-related preferences, by sacrificing fluid to view images of high-status, but not low-status monkeys (Deaner, Khera, & Platt, 2005). However, an attempt to determine whether female rhesus macaques visually distinguish facial “masculinity” from varying face morphology of males revealed borderline results (Rosenfield et al., 2019). Similarly, assessment of whether brown capuchins differentiate between faces of varying facial width found that subjects did not differ in their approach latency to a life-sized capuchin model with manipulated facial image depicting lower or higher fWHR (Wilson, Gartner, D’Eath, Buchanan-Smith, & Morton, 2018). However, it was unclear whether their response was because they did not use fWHR as a cue to Assertiveness or as a result of viewing static stimuli rather than live conspecifics.

Alongside behavioural paradigms, studies from neuroscience can also address this question. For example, in response to superior compared with inferior co-players, human participants show increased activation in the occipital/parietal cortex, ventral striatum, parahippocampal cortex and dorsal lateral prefrontal cortex (Zink et al., 2008). This overlaps with findings of responses to social cues of status, such as direct gaze and brow position (Marsh, Blair, Jones, Soliman, & Blair, 2009). Notably, men with higher fWHRs had increased amygdala activation in response to images of angry facial expressions (Carré, Murphy, & Hariri, 2013). Lesions to the amygdala in rhesus macaques reduced affiliative tendencies in response to threats from conspecifics compared with pre-lesion baseline (Machado & Bachevalier, 2006). This raises the possibility that emotional reactivity could explain the relationship between facial features and dominance. Given the likely differential expression of such behaviour between species and social organisations, this could also explain the wide variance in reported findings to date. Whilst these studies examine response to behavioural, or perceived behavioural information, approaches that use EEG or fMRI could be useful in understanding social perceptions of static facial morphology.

It is clear that more research into the perception of personality and facial morphology in nonhuman primates is needed. Whilst the looking time paradigm can provide a baseline for understanding whether a study group can distinguish such information, this approach can have limited interpretation in understanding the social value of these features (Morton et al., 2016; Wilson et al., 2023). This approach should therefore be complemented not only by a neuroscience perspective but also by studies that try to understand social outcomes of sexually dimorphic facial features in natural settings.

## 6. Future directions

In this manuscript, we have drawn on literature addressing the behavioural and neuroendocrinological correlates of facial morphology within an evolutionary framework. By doing so, we hope to have highlighted the benefits of comparative research for understanding how, and why, the primate face can cue personality traits. Here we highlight some specific steps that would be of benefit to the field:

Firstly, we should move away from single measures such as the fWHR and measures based on ratios (due to spurious correlations; Kronmal, 1993) and consider facial morphology holistically (Dixson, 2018; Kleisner, Pokorný, & Saribay, 2019). This would require taking multiple measures (e.g., sexual shape dimorphism, averageness, Kočnar et al., 2019) based on the configuration of facial landmarks delineating facial shape, ideally from both skulls and from flesh (or non-invasive proxies of either) (Fig. 1c). Moreover, diverse facial ornamentation (such as the presence of ear or head tufts, beards or other facial hair) evolved across the primate taxa (Petersen & Higham, 2020). Despite extensive research suggesting the potential role of human facial hair in signalling dominance (Dixson & Vasey, 2012, but see also Kowal et al., 2021), the link between shape and size variation of primate facial hair features and behavioural variation has not been studied yet. Future studies thus should explore the potential facial hair-personality links and their selection pressures (Dixson et al., 2005; Petersen & Higham, 2020).

Secondly, we need to better understand the proximate mechanisms underlying facial-personality links in primates. Such research could benefit from in-depth consideration of developmental neuroendocrine mechanisms, as well as other possible latent variables such as emotional reactivity. Laboratory colonies of primates represent a viable research opportunity due to their experience in neuroendocrine, genetic and developmental research. Later, expanding the research beyond fWHR measures and beyond the laboratory to a broad spectrum of primate species with varying social and mating strategies and relatedness levels would help shed light on proximate as well as ultimate facial-personality links in primates, including humans.

Thirdly, we can utilise a comparative understanding of the role of faces in social cues or signals, by drawing comparisons amongst phylogenies of varying socio-ecology. Specifically, more studies on personality links to reproductive success (Brent et al., 2014; Masilkova, Boukal, Ash, Buchanan-Smith, & Konečná, 2022; Weiss et al., 2023) and the importance of personality in mate choice are needed. Studies that not only incorporate populations from western, educated, industrialised, rich and democratic populations but also people from indigenous communities could better aid the understanding of such selection pressures in humans.

To conclude: does the primate face cue personality? The answer appears to be yes, but we are only just beginning to understand how and why.

## Footnotes

1
*A **signal** provides information of interest to the receiver, which evolved because of its effect on the receiver. A **cue** provides information to a receiver, but is not necessarily intended for the receiver; that is, it did not evolve because of its effect on the receiver (Maynard Smith, & Harper 2003)*.

## Supplementary material

For supplementary material accompanying this paper visit https://doi.org/10.1017/pen.2023.5. click here to view supplementary material
