# Supplementary material for: Does the primate face cue personality?
Source: Personal Neurosci. 2023 Aug 9;6:e7. doi: 10.1017/pen.2023.5 (PMC10725780; doi:10.1017/pen.2023.5)
Supplement: Supplementary file 1 [file S2513988623000056sup001.docx]

| **Number of landmark** | **Type of landmark** | **Definition** |
| --- | --- | --- |
| 1 | L | midpoint of upper line of head |
| 2 | L | midpoint of mandible |
| 3, 4 | L | inner corner of eye fissure where eyelids meet |
| 5, 6 | L | outer corner of eye fissure where eyelids meet |
| 7, 8 | L | the highest point of eyelid |
| 9, 10 | L | the lowest point of eyelid |
| 11, 12 | L | inner corner of nostril |
| 13, 14 | L | the most lateral point of nostril |
| 15 | L | midpoint of nose between the highest points of nostrils |
| 16 | L | midpoint on the line between the corners of the eye |
| 17 | L | midline point and the highest point of upper lip |
| 18, 19 | L | outer corner of mouth where outer edges of upper and lower lip meet |
| 20, 21 | S | midpoint between 17 and 18 and between 17 and 19 |
| 22, 23 | L | the widest point of the zygomatic arch |
| 24, 25 | L | the widest jaw edges |
| 26, 27 | S | midpoint between 22 and 24 and between 23 and 25 |
| 28, 29 | S | midpoint between 18 and 2 and between 19 and 2 |
| 30 | L | midpoint of upper line of brow ridge |

Supplementary material for:

**Does the primate face cue personality?**

Wilson, V. A. D. & Masilkova, M.

*Personality Neuroscience*

__________________________________________________________________________________

**Table S1.** List of 30 facial landmarks and semi-landmarks for holistic facial morphometric measurements in primates.

*Note:* L = landmark, S = semi-landmark.
